# Diagnosis of COVID-19 with simultaneous accurate prediction of cardiac abnormalities from chest computed tomographic images

**DOI:** 10.1371/journal.pone.0290494

**Published:** 2023-12-14

**Authors:** Moumita Moitra, Maha Alafeef, Arjun Narasimhan, Vikram Kakaria, Parikshit Moitra, Dipanjan Pan

**Affiliations:** 1 Center for Blood Oxygen Transport and Hemostasis, Department of Pediatrics, University of Maryland Baltimore School of Medicine, Baltimore, Maryland, United States of America; 2 Department of Chemical, Biochemical and Environmental Engineering, University of Maryland Baltimore County, Baltimore, Maryland, United States of America; 3 Biomedical Engineering Department, Jordan University of Science and Technology, Irbid, Jordan; 4 Department of Nuclear Engineering, The Pennsylvania State University, State College, Pennsylvania, United States of America; 5 Department of Materials Science & Engineering, The Pennsylvania State University, State College, Pennsylvania, United States of America; 6 Huck Institutes of the Life Sciences, State College, Pennsylvania, United States of America; Jordan University of Science and Technology, JORDAN

## Abstract

COVID-19 has potential consequences on the pulmonary and cardiovascular health of millions of infected people worldwide. Chest computed tomographic (CT) imaging has remained the first line of diagnosis for individuals infected with SARS-CoV-2. However, differentiating COVID-19 from other types of pneumonia and predicting associated cardiovascular complications from the same chest-CT images have remained challenging. In this study, we have first used transfer learning method to distinguish COVID-19 from other pneumonia and healthy cases with 99.2% accuracy. Next, we have developed another CNN-based deep learning approach to automatically predict the risk of cardiovascular disease (CVD) in COVID-19 patients compared to the normal subjects with 97.97% accuracy. Our model was further validated against cardiac CT-based markers including cardiac thoracic ratio (CTR), pulmonary artery to aorta ratio (PA/A), and presence of calcified plaque. Thus, we successfully demonstrate that CT-based deep learning algorithms can be employed as a dual screening diagnostic tool to diagnose COVID-19 and differentiate it from other pneumonia, and also predicts CVD risk associated with COVID-19 infection.

## Introduction

Coronavirus disease (COVID-19) that emerged in December 2019 in Wuhan, China, is a severe respiratory illness that has caused millions of deaths around the world [1]. Most individuals infected with COVID-19 causative SARS-CoV-2 virus will experience mild to moderate respiratory symptoms [2], but often, this infection can proceed to severe pneumonia, multi-organ failure, or even death. Scientists, therefore, tried to distinguish this deadly disease from regular influenza-caused diseases, however, differentiation among different pneumonia cases including SARS-CoV, MERS-CoV, and influenza-related pneumonia is still a challenge.

Interestingly, SARS-CoV-2 is also known to infect the cardiovascular system [3]. Although primarily a respiratory disease, acute cardiac damage has been found as a common extrapulmonary manifestation of COVID-19 with potential detrimental significance. Older people, as well as those who have underlying medical conditions such as cardiovascular disease, diabetes, and cancer, are more likely to acquire serious illnesses [4, 5]. Myocardial infarction, myocarditis, heart failure, and arrhythmias can happen when infected by this virus [6, 7]. Cardiac enlargement has also been observed from CT images of COVID-19 patients [8, 9].

Till date, reverse-transcription polymerase chain reaction (RT-PCR) testing and chest computed tomographic (CT) imaging are the only established tools for the diagnosis of COVID-19 [10–18]. Some emerging point-of-care diagnostic tests have been proposed for the selective diagnosis of COVID-19 [19–27], however, in patients with negative RT-PCR tests, only CT can diagnose COVID-19 at an early and asymptomatic stage [28, 29]. Despite having these extraordinary capabilities, differentiating COVID-19 from other pneumonia cases, and accurately predicting the associated cardiac abnormalities from the same CT scan image is quite an ambitious assignment that is not explored yet. AI-based image analysis techniques prove their ability to diagnose different diseases including pneumonia from chest CT and X-ray images [30, 31]. Several machine learning, particularly deep learning (DL) mostly convolutional neural networks (CNN), have been shown to have an extraordinary performance for automatic diagnosis of COVID-19 from clinical CT scan and chest X-Ray images [32–37]. Transfer learning models have also been found to be a viable option and widely implemented in many recent studies. In one of the studies a deep architecture was proposed to identify COVID in CT images. They have used the feature extractor VGG16 and the principal component analysis to choose among the feature that were extracted. For classification, they used the bagging method, Support Vector Machine, and Extreme Learning Machine. Three datasets, specifically those gathered from the Italian Society of medical and Interventional research, were used to examine the methodology. This method achieved 95.7% accuracy [38]. One of the other studies proposed a transfer learning method using VGG19 model [39]. They worked on a small dataset and the proposed method achieved only 84% accuracy for classifying COVID-19 CT scan images, while other existing deep learning models perform poorly. Other studies also reported an automated COVID-19 classification using different transfer learning models and compared their performances [40–44]. Asad et al. recently used an ensemble method with Efficientnet pretrained model to detect COVID-19 from Chest X-Ray images [45]. Ahmed et al. recently proposed a hybrid and CNN ensemble model to detect COVID-19 from CT scan images [46]. They have used MobileNetV2 with CNN model. In both cases, they used COVID-19 infected and uninfected healthy images. Sometimes other pneumonia infection can also be present, and it looks similar to the COVID-19 infection. Identification of other pneumonia and classify them from COVID-19 is also an important factor. At the same time, all these DL approaches are trained with a large number of datasets. However, due to a shortage of images, creating a big, annotated data set for every disease is not feasible.

Our primary focus was to identify CVD specifically related to COVID-19 infection. In our study, we employed three established parameters, namely Cardiac Thoracic Ratio (CTR), Pulmonary Artery to Aorta Ratio (PA/A), and quantification of epicardial adipose tissue and the presence of calcium plaque, as biomarkers that indicate CVD. These parameters have been supported by previously published independent studies as indicators of CVD. Yotsueda et al. found a correlation between an increased CTR and higher risks of heart failure and other cardiovascular events [47]. Karakus et al. demonstrated a link between an increased PA/A ratio and pulmonary hypertension in patients with specific heart valve disease [48]. Additionally, Eslami et al. demonstrated that CT-measured cardiac indices are associated with the severity of lung involvement and can predict survival in COVID-19 patients [4]. They performed clinical studies and observed abnormal cardiac structure for COVID-19 infected patients as compared to the normal patient. This study shows that lung involvement CT scores are positively correlated with elevated CTR and increased PA/A. Zheng et al. [49] and Guzik et al. [50] recently showed that Angiotensin-converting enzyme 2 (ACE2) is a membrane-bound aminopeptidase that has a vital role in the cardiovascular and immune systems. ACE2 is involved in heart function and the development of hypertension and diabetes mellitus. In addition, ACE2 has been identified as a functional receptor for coronaviruses, including SARS-CoV and SARS-CoV-2. SARS-CoV-2 infection is triggered by the binding of the spike protein of the virus to ACE2, which is highly expressed in the heart and lungs. However, this information is observed from clinically researched datasets. Computerized automated technology for the detection of cardiovascular abnormalities along with COVID-19 has not been proposed, as professional radiologists are still required to make the diagnosis [51]. Further, the Evaluation of the risk of developing CVD complications has recently been shown to provide a wealth of information about the survival of COVID-19 patients. Therefore, the need of the moment is a viable and user-friendly AI-based platform for not only differentiating COVID-19 from other pneumonia cases but also, automatically detecting cardiac abnormalities from the same CT images of infected individuals. Thus, we propose herein an end-to-end AI-based technique to solve 1) the difficulty in classifying COVID-19 pneumonia from non-COVID19 causative pneumonia and healthy individuals using a smaller dataset with high accuracy 2) simultaneously recognize the risk of developing CVD complications associated with COVID-19.

To differentiate COVID-19 from healthy and other non-COVID-19 causative pneumonia, the classification was performed using pre-trained models as feature extractors with ML classifiers to obtain a high accuracy level. Transfer learning is a CNN-based deep learning approach that is an automated form of standard machine learning in which the system determines which features to employ. Learning features from DL models and utilizing them to train another ML model requires less computational resources and time. In our study, we have explored other transfer learning models as deep feature extractors and supervised machine learning models for classification. At the same time, we validated our entire data with the K-fold cross-validation technique. This study also investigated the accuracy of existing pre-trained models and the proposed hybrid model. Further, we developed a robust deep learning model that can predict the CVD risk from the CT scan of COVID-19 patients rapidly and efficiently.

**Fig 1:**
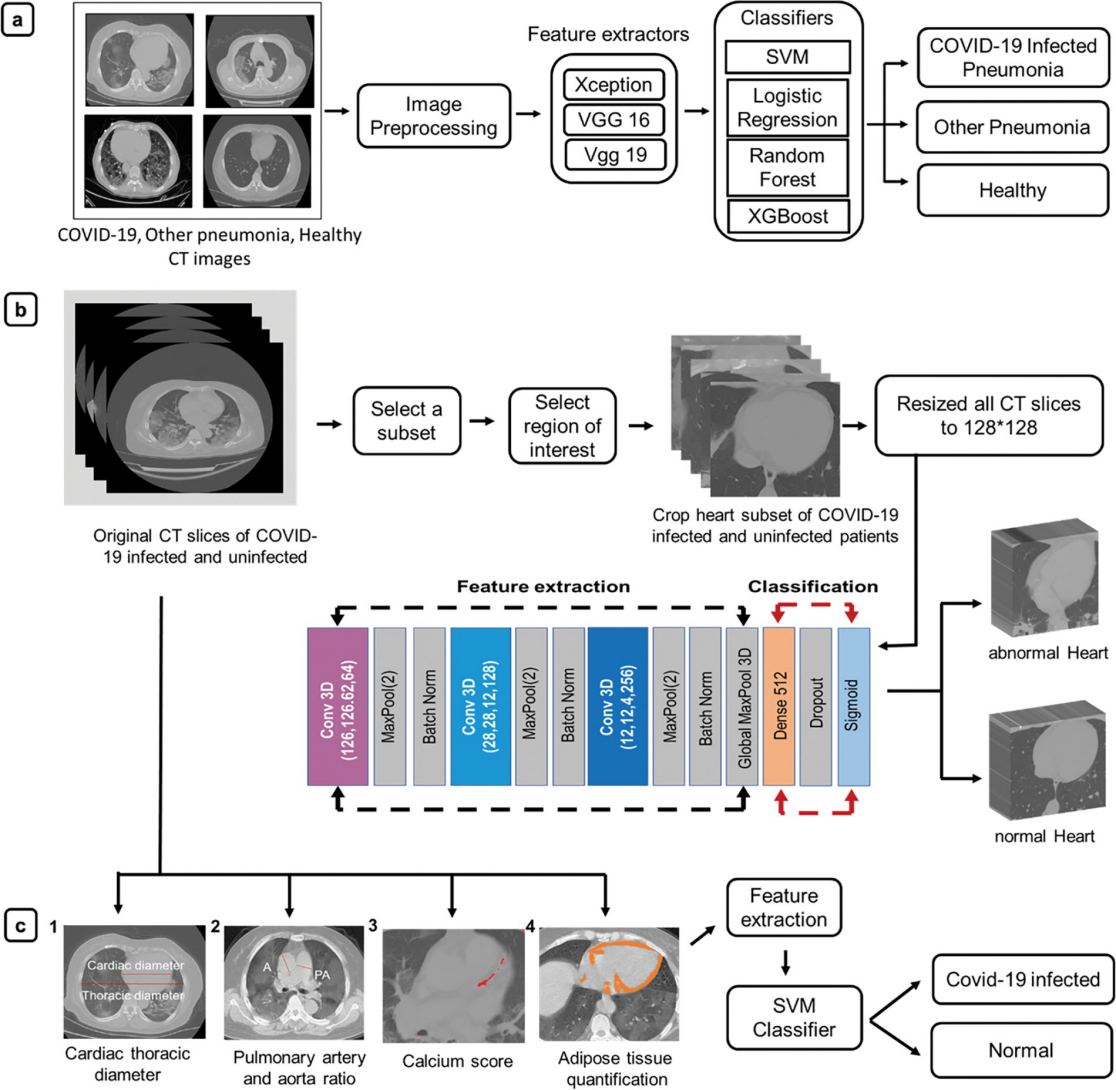
(a) Classifying COVID-19 pneumonia from non-COVID-19 cases: COVID-19 infected, other pneumonia infected, and uninfected CT images were classified using three different pre-trained models VGG16, VGG19 and Xception, with ML classifiers, SVM, Random Forest, Logistic regression, and XGBoost (b) Prediction of cardiac abnormalities in COVID-19 infected patients: 3D-CNN model consists of three convolution layers followed by ReLU, Maxpool layer, batch normalization layer for feature extraction from the cardiac region, with the classification part consists of one dense layer, ReLU, dropout layer, and activation function sigmoid. Finally, it diagnosed abnormal cardiac health from CT-scan of COVID-19 infected and healthy subjects. (c) Classification of cardiovascular abnormalities using CT-measured cardiac parameters. Cardiac parameters were extracted from the 3D CT slices of COVID-19 infected patients and uninfected patients. The parameters were 1) cardiac and thoracic diameter ratio, 2) pulmonary artery and aorta ratio, 3) presence of calcified plaque and 4) mean epicardial adipose tissue area. ML classifier support vector machine (SVM) was then used to predict the impact of COVID-19 on cardiac health from CT measured parameters.

To the best of our knowledge, this work is the first automated approach for predicting the cardiac abnormalities associated with COVID-19 using chest CT images. In this work, we proposed and successfully demonstrated the use of a 3D CNN model for a PC-aided prediction of cardiovascular complications using a chest CT. Our platform provides a robust, accurate, and cost-efficient tool for the diagnosis of cardiac abnormalities associated with COVID-19 infection. Overall, in this work, we developed a dual-screening quantitative tool to selectively diagnose COVID-19 separating it from other non-COVID-19 causative pneumonia and healthy cases and simultaneously predicting the COVID-19 associated cardiac abnormalities. The purpose of this study was not to conduct a prospective clinical study and collect data, but rather to develop ML tools using publicly available data. Our model exhibited promising results in all conducted experiments. The developed dual-screening diagnostic tool may be successful in assessing COVID-19 with primary data, even though our findings are based on secondary data. This study provides valuable insights into diagnosing COVID-19, separating it from other pneumonias, and predicting CVD risk associated with infection. Thus, this study will help to build up a new AI-based user-friendly system for simultaneous classification and accurate prediction of cardiac anomalies in COVID-19 infected patients from a single CT scan. **Fig 1** depicts a schematic representation of the workflow used for the diagnosis of COVID-19 and predicting the associated CVD complications. Representative CT images of COVID-19 associated CVD risk estimated subjects (**S1 Fig in S1 File**) and healthy subjects (**S2 Fig in S1 File**) were shown in the supplementary information.

## Methods

The contribution of this research entails the following points: (1) First we designed a deep learning-based classification model consisting of a pre-trained model and classifiers to distinguish COVID-19 from other pneumonia and uninfected subjects; (2) After that a 3D-CNN model has been designed to predict cardiac abnormalities from 3D cardiac volumetric images and (3) Cardiac parameters such as cardiac and thoracic diameter ratio, pulmonary artery and aorta ratio, presence of calcified plaque and mean epicardial adipose tissue area has been measured for COVID-19 positive and negative subjects and analyzed the impact of COVID-19 on the cardiac system using ML classifiers.

### Pretrained models with ML classifier for classification of non-COVID-19 pneumonia cases from COVID-19

In this section, we described some of the existing CNN-based deep pre-trained models as feature extractors and ML classifier to classify COVID-19 from other pneumonia and healthy subjects using CT images.

#### VGG16 and VGG19

Karen Simonyan and Andrew Zisserman of the Oxford Robotics Institute created the Visual Geometry Group Network (VGG) using a convolutional neural network design. It was addressed at the 2014 Large Scale Visual Recognition Challenge (ILSVRC2014). It is well-known that the deeper the network, the better the performance. This explains the superior performance of VGGNet as a deep network that has twice the number of layers as the commonly used CNN. VGGNet worked very well on ImageNet dataset. To enhance picture extraction efficiency, the VGGNet used smaller filters of 3×3. This deep network includes two types: VGG16 and VGG19, each with its own set of depths and layers. The use of VGGNet for feature extraction originated from its superior performance, what makes this model unique when compared to other vision models is the implantation of convolution layers of 3x3 filter and a convolutional stride of 1 and consistently using maxpool layer of 2x2 filter and stride value of 2 as well as same padding. The excellent performance of VGGNet when combined with another ML classifier may be attributed to the huge number of extracted parameters (approximately ~138 million).

#### Support vector machine

Support Vector Machine is the most extensively utilized state-of-the-art machine learning approach. Support-vector machines are supervised learning models with related learning algorithms used in machine learning to examine data for classification and regression analysis. SVM’s binary classifier produced either positive or negative results. The multi-class binary classifier was later merged to improve it. Furthermore, the SVM can be utilized to plot the input space into nonlinear instances. By using maximal hyperplanes in SVM, the input feature can be transferred to a higher feature dimension. It is possible to improve accuracy by using kernel and hyperparameters. The kernel parameters are essential for optimizing data and determining the optimum model.

#### Proposed network

To implement the proposed hybrid classification model, we have used a transfer learning approach which consists of a pretrained model as feature extractor with a ML classifier [52]. The pretrained model extract the unique features from the data that indicate distinct opacity regions in the lungs, as well as how much they differ between COVID-19 infected, other pneumonia infected, and healthy people. Those extracted features are flattened vectors for each input image that encapsulates these high-level features, used as input to train a ML classifier to distinguish COVID-19 infected patients from healthy and uninfected subjects.

**Fig 2:**
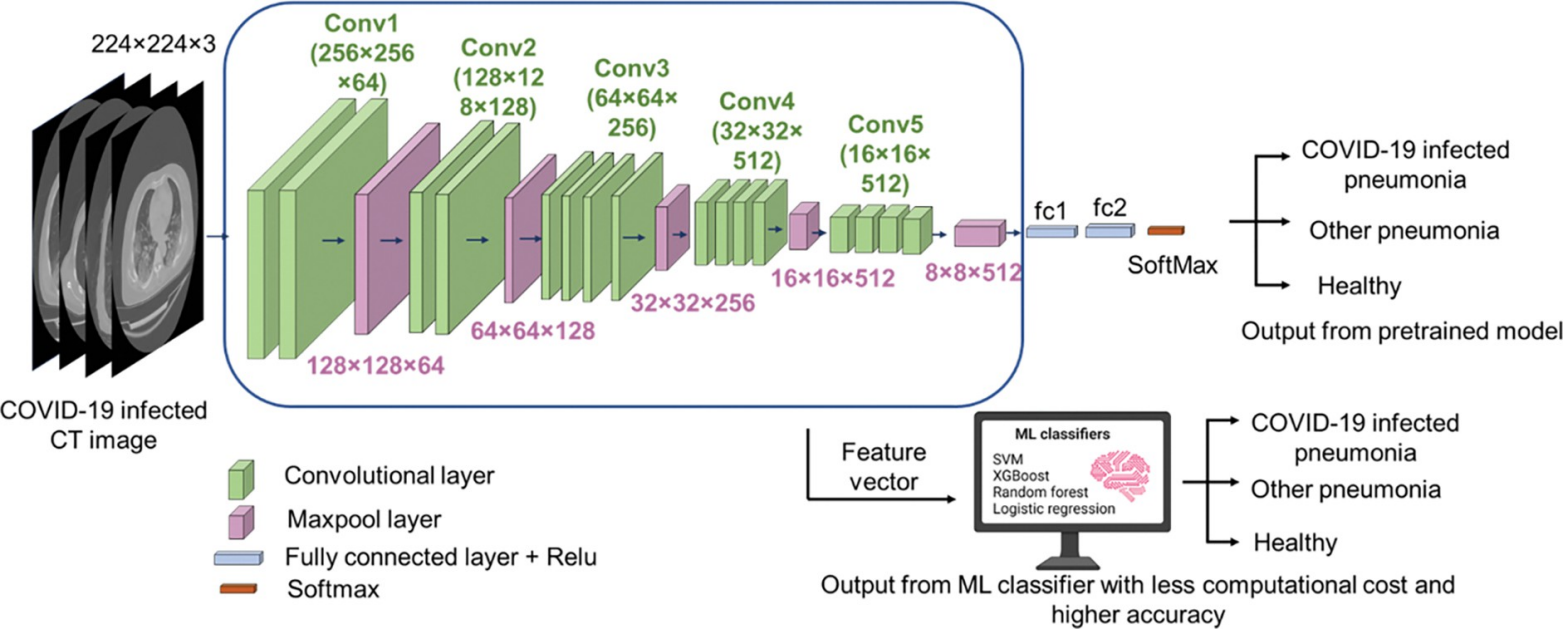
Network architecture for the proposed method. Initially, pre-trained model VGGNet has been used as the most significant CNN base deep feature extraction technique, which is subsequently fed into supervised ML models for the final representation of a proposed hybrid model.

Transfer learning process begins with a pretrained model that has been trained on millions of images from the ImageNet database. These pre-existing models (VGG16, VGG19, Xception etc.) have already been learn a vast number of intricate patterns, therefore it is proficient to extract meaningful features from input images. During feature extraction phase, the final output layer of the pre-trained model is replaced with layers suitable for our specific task. The layer from pre-existing model kept frozen to maintain the weights and the new classification layers added to train on the new dataset. This way, we use the pre-trained model to extract useful features and train a classifier on top of these features for our specific task. During the training phase, ML classifiers uses the feature vectors and their corresponding labels. After training, those feature vectors from new images (extracted using pre-trained model) were fed into the ML classifier to predict their classes, based on that it can distinguish input images into different classes. All the CT slices were resized to 224×224 to use as inputs. Applied one-hot encoding on the labels of image data to identify different classes. In our study we have used multiple transfer learning models and examined their performance. Pretrained CNN models, VGG-16, VGG-19, and Xception were used as feature extractors with the ML classifiers support vector machine (SVM), random forest (RF), logistic regression (LR), and XGBoost (XGB). The architecture of pre-trained model VGGNet with ML classifiers has been shown in **Fig 2**.

### Development of 3D-CNN model for the prediction of cardiac abnormalities in COVID-19 infected patients

**Fig 3:**
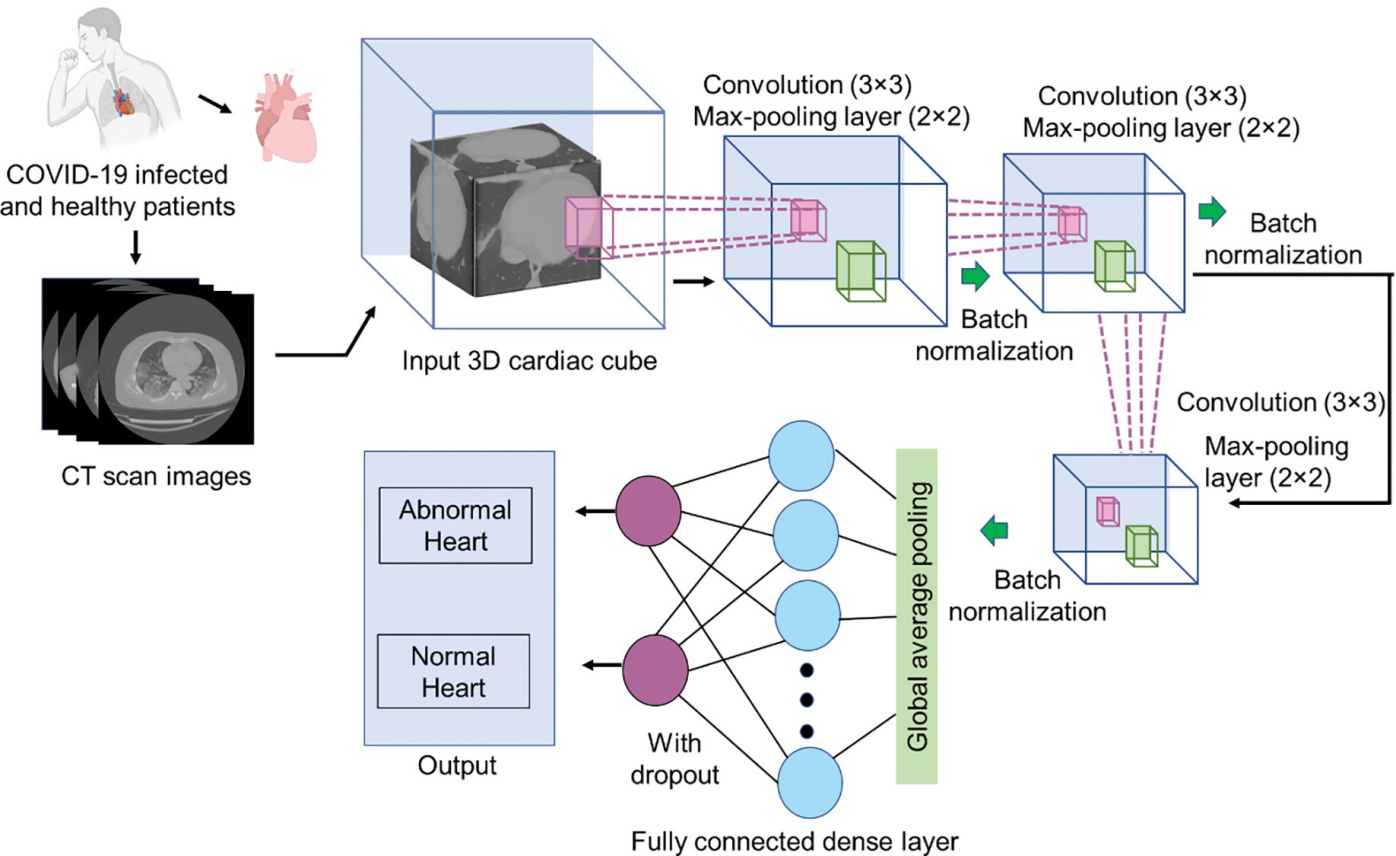
Flowchart of proposed 3D-CNN model applied to detect COVID-19 associated cardiac abnormalities from CT scan images.

To conduct the CVD classification, we utilized a dataset consisting of 162 COVID-19-infected subjects and 167 healthy individuals. Patients’ severity distribution in our dataset has been represented in a spreadsheet table (**S4 Table in S2 File**). It is worth noting that the majority of patients within the COVID-19-infected group exhibited moderate to severe conditions as shown in **S9a Fig (S1 File)**. Original 3D CT scans were collected from publicly available datasets. 3D cardiac volume from the original CT scan was used as an input to CNN’s deep classification pipeline that comprised the feature extraction and classifier components to classify the CVD-associated COVID-19 patients from healthy subjects. A thirteen-layer 3D-CNN architecture has been developed with each convolution layer with kernel size 3×3, each max-pool layer with kernel size 2×2. The feature extraction part consisted of three convolutional (CONV3D) layers. Each CONV3D layer contained the activation function ReLU which was followed using a MaxPool layer and a batch normalization (BN) layer. Here we used adam optimizer [53]. Three CONV3D layers were thus comprised of a single layer with 64 filters followed by 128 and 256 filters. Three Maxpool layers were used with each containing a pool size of two. All the filters were set with a kernel size of 3×3×3. All features were extracted from the global average pooling layer. The output from the feature extraction part was flattened and transferred into a fully connected dense layer. A fully connected dropout layer was added to avoid overfitting [54]. The output was then transferred to a dense layer with an activation function sigmoid for binary classification [55]. The architecture of 3D-CNN model has been shown in **Figs 1B and 3**. The model had a total of 1,241,089 trainable parameters, which classified 99 independent test images, with 97.97% accuracy. Here we considered keeping the network relatively simple to avoid overparameterization problems as only a few numbers of training samples were used [55]. Our deep CNN model was implemented in Python using TensorFlow and the model was trained for 23 epochs on NVIDIA corporation VGA compatible controller (GK106GL [Quadro K4000]) running on Intel(R) Xeon(R) CPU E5-2680 v2 @ 2.80GHz with 20 processors.

### ML pipeline to evaluate the impact of COVID-19 on cardiac health

Four selected cardiac parameters, i.e., CTR, PA/A, CAC and mean epicardial adipose tissue area, were calculated in all 329 CT images of COVID-19 infected and uninfected subjects (**Fig 1C, S4 Fig in S1 File**). On axial CT images, the CTR was determined by dividing the largest transverse cardiac diameter measured from outer-to-outer myocardium by the biggest transverse thoracic diameter calculated from the inner-to-inner chest wall (**S5 Fig in S1 File**). A CTR of greater than 0.49 is considered as a sign of cardiac hypertrophy [56]. For calculation of PA/A, the PA was measured at the level of bifurcation to the ascending diameter of the aortic from the same CT slice (**S6 and S12 Figs in S1 File**). Enlargement of PA is evident when PA/A is greater than 1 and can be attributed to pulmonary hypertension [48]. The abundance of calcium plaques is also considered as cardiac abnormality (**S7 Fig in S1 File**) [4, 57]. Mean epicardial adipose tissue area was calculated for three slices of each 3D CT scan for all COVID-19 infected and uninfected patients. Three slices were chosen from the beginning, middle, and end of the cardiac component in a CT scan (**S8 Fig in S1 File**). All these calculated CT measured data sets were represented in a spread sheet supplementary table (**S4 Table in S2 File**). These parameters were used as input for ML classification. The training set was used to train the model and then implemented on a separate test set. AUC (threshold independent parameters) score was calculated to optimize the model performance. Threshold-dependent parameters (sensitivity, specificity, and accuracy) were also calculated while evaluating the model with the test set [58]. The K-fold cross-validation method was then used over the entire data and optimized the model to avoid over-fitting and obtain better generalization ability.

## Results and discussion

### Datasets

This study is based on a publicly accessible CT scan database (database links are provided below in the data availability section) of COVID-19 patients diagnosed using the gold standard RT-PCR test. COVID-19 infected, and uninfected CT scans was collected from the Cancer Imaging Archive (TCIA) COVID-19 datasets, RSNA and MosMed and Rediopaedia databases. A machine learning-based classification was first developed to diagnose COVID-19 pneumonia compared to other pneumonia and healthy subjects. We have used a total of 415 CT images including, 162 COVID-19 infected, 167 uninfected healthy subjects and 86 CT scan images for other pneumonia-infected patients were collected from medical image data resource center (MIDRC) datasets to include in this study. Other-pneumonia infected images were comprised of cases from MERS-CoV, SARS-CoV, TB and influenza (**S9b Fig in S1 File**). A selection of representative images for SARS, MERS, and viral pneumonia has been provided in **S3 Fig (S1 File)**. Although in most of the case patient’s demographic information’s were no provided due to patient’s privacy and ethical regulations, but for some cases databases were available with information’s like age, gender and type of disease. That information’s were represented in a spreadsheet (**S5 Table in S3 File**). To develop the 3D-CNN model for the prediction of cardiac abnormalities in COVID-19 patients compared to the healthy subjects, the same CT scans of 162 for COVID-19 infected and 167 for healthy subjects, were used. CT scans are of different slice spacing varying from 5 to 8 mm. The subjects were randomly divided into training and test sets. To perform CVD screening on this dataset, we labeled each subject as either CVD-positive or CVD-negative. The subjects were further considered as CVD positive based upon the CT measured gold standard cardiac parameters including CTR, PA/A and observed calcified plaques. COVID-19 infected CT images associated with these cardiac anomalies were assessed as carrying higher CVD risk. In our study, we have used a combined dataset collected from different publicly available data. This has helped in increasing the robustness and generalization capability of the proposed method to evaluate the clinical images from a wide range of sources.

### Differentiating COVID-19 associated pneumonia from non-COVID-19 causative pneumonia

Herein we have considered multiple CT slices of a 3D CT scan as inputs in different pre-trained CNN models for feature extractions with ML model for classifying COVID-19 and differentiating it from other pneumonia and uninfected healthy cases [59]. A total dataset of 415 CT scans was collected from 162 COVID-19 infected patients, 86 non-COVID related-pneumonia infected patients and 167 uninfected healthy subjects. We evaluated the test dataset with different feature extractor pre-trained models with ML-classifiers and observed the accuracy.

#### Model evaluation

To investigate the performance of the developed model, k-fold cross-validation has been used. The main reason for using k-fold cross-validation is that it is an unbiased indicator and gives a proper representation of the model performance [60]. In addition, the root means square error (RMSE) is not a beneficial estimator of model performance in the conventional validation and therefore the assessment of model performance could not be adequately represented by the error of the test data set [61]. In the k-fold validation, the original sample is randomly partitioned into k equal sized subsamples. Here, as validation data for testing the model, one of these k subsamples was used, whereas the k—1 subsamples were used for the model training. The mean squared error, MSE_1_ then computed on the observations of the held-out fold. This process then repeated k-folds or times to assure that each of the k subsamples was employed once to validate the model. The final estimate is thus a single value that can be calculated by averaging the k estimates of the test error *MSE*_1_,……*MSE_k_*, as per the equation mentioned below. The selection of the folds was such that the mean response value was approximately equal in all the folds in the stratified k-fold cross-validation model [61, 62]. Classification results were evaluated using accuracy, Receiver Operating Characteristics (ROC) curve and the confusion matrix. All these measuring parameters are computed as follows.


$${CV}_{\left(K\right)}=\frac{1}{K}\sum_{i=0}^{K}{MSE}_{i}$$

$$Accuracy=\frac{true\;positive+true\;negative}{true\;positive+true\;negative+false\;positive+false\;negative}$$

Where true positive is the number of correctly detected positive cases, false positive is the number of negative cases incorrectly detected as positive, false negative is the number of positive cases incorrectly detected as negative, and true negative is the number of negative cases, correctly not detected.

#### Model prediction scores and result comparison

**Fig 4:**
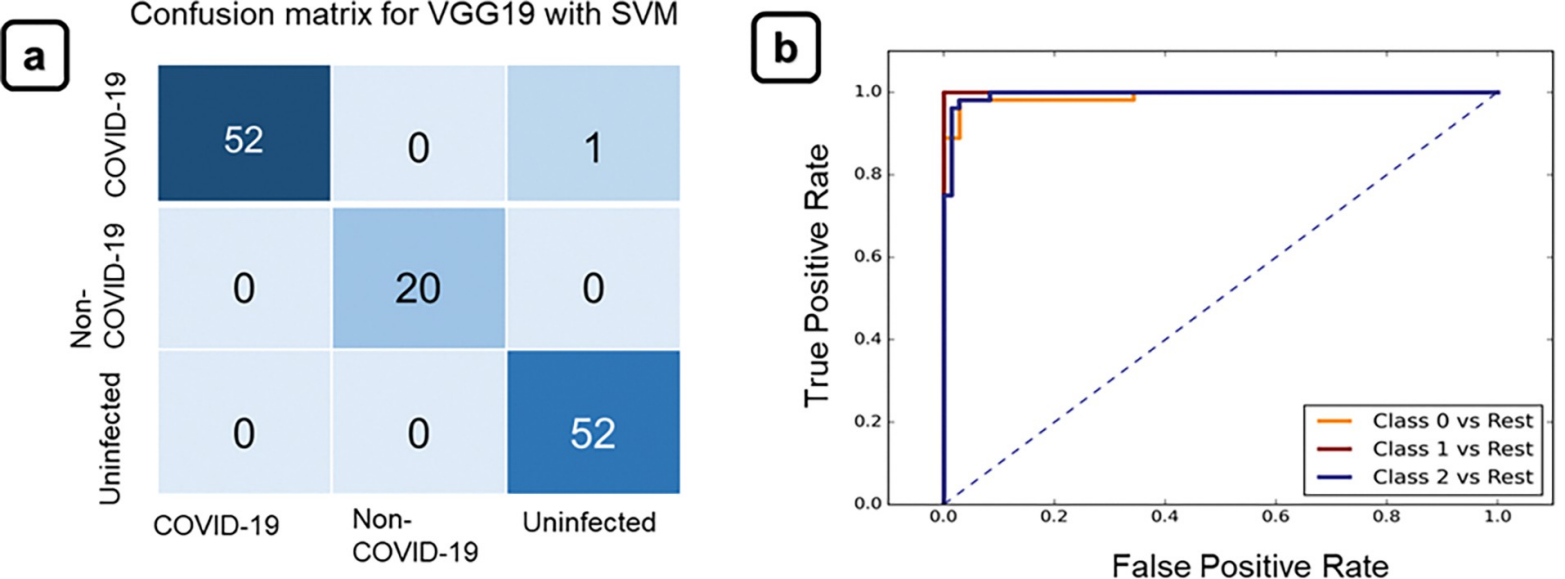
(a) Confusion matrices and (b) ROC curve for multiclass classification with pre-trained model VGG19 and machine learning classifiers SVM, showing the performance of classification on independent test data set. Images classified into three classes of COVID-19 pneumonia, other pneumonia, and healthy subjects.

It was observed that the pre-trained model, i.e., VGG19 with ML classifier SVM and logistic regression (LR) achieved the highest accuracy of 99.2% to classify the data into three classes, COVID-19 pneumonia, non-COVID-19 related pneumonia, or uninfected healthy cases. Accuracy results for all the above models were evaluated by partitioning the data into 70% training and 30% independent testing datasets. On the independent test set, VGG19 with ML classifier XGBoost and random forest obtained 96% and 97.6% of accuracy respectively. On test set VGG16 with ML classifier SVM, LR, XGBoost and random forest obtained 98.4%, 98.4%, 97.6% and 96.8% of accuracy respectively. Similarly, on an independent test set Xception with ML classifier SVM, LR, XGBoost and random forest has obtained 96.8%, 96.8%, 96% and 97.4% accuracy respectively. We also checked the performance using the CNN-based pre-trained models for classification. In that case pre-trained model, Xception achieved the highest accuracy 96.8%. However, the performance of our proposed hybrid model which is ML classifier with extracted deep features has outperformed the other models in terms of accuracy as well as both sensitivity and specificity metrics from below mentioned formula. At the same time, the training time and overall execution time of the hybrid model were faster as compared to the classification and detection performed by pre-trained models. Computational training and execution time for all models are represented in **Table 1.** The memory size consumed by the proposed hybrid model is 787217 bytes and for the pre-trained model it is 81026430 bytes. This shows that the proposed model has a lower computational cost and is more efficient. The confusion matrix for the best-performed model is represented in **Fig 4A** and the confusion matrix for all the other models is represented in **S10 Fig (S1 File)**. Accuracies, sensitivity, and specificity for a single dataset for all models are represented in a heatmap (**S2 Table S1 File**). Model evaluation using K-fold cross-validation has also been performed. VGG19 and VGG16 with ML classifier SVM and LR achieved the highest average cross-validation accuracy of 98.5%. As a feature extractor performance, VGG19 and VGG16 were found to behave similarly [63]. The average cross-validation score of VGG19 and VGG16 with ML classifier XGBoost and the random forest was 97.3% and 97.6%. Other models like Xception with SVM, logistic regression, XGBoost and random forest achieved 97.6%, 97.3% and 95.6% and 96.6% average cross-validation accuracy, respectively. A 10-fold cross-validation ROC curve for the VGG19 with SVM classifier was thus constructed and represented in **Fig 4B**. All the average 10-fold cross-validation accuracy data has been reported in (**S1 Table in S1 File**). From the confusion matrix, we can see the number of misclassified images for each model. Confusion matrix for VGG19 with SVM model showed one misclassified covid-19 image as uninfected. Misclassified image has slight inflammation in the cardiac region, but low opacity region and our model detected that COVID-19 infected CT image as the normal healthy subject. Thus, this study is showing the performance of different ML pipelines and confirms that an ML pipeline can successfully be developed to classify COVID-19 patients from non-COVID-19 related pneumonia and healthy individuals. Misclassified image has been represented in **S11 Fig in S1 File**.


$$Sensitivity=\frac{Predicted\;COVID-19\;images}{Total\;COVID-19\;images}$$

$$Specificity=\frac{Predicted\;non\;COVID-19\;images}{Total\;non\;COVID-19\;images}$$

**Table 1:** Computational time and execution time for pretrained model and proposed hybrid model.

Classification models	Training and execution time (seconds)
**VGG16**	300.00
**VGG19**	370.00
**Xception**	270.00
**Hybrid model**	115.00

### Prediction of COVID-19 associated CVD from CT scans

Developing a dual screening diagnostic tool for the diagnosis of COVID-19 and identifying the associated CVD risk is still a significant challenge, with negative consequences to the patient’s life as well as the health care system. The capability of our system to differentiate COVID-19 pneumonia from other non-COVID-19 causative pneumonia and healthy subjects has been established. This motivates us to investigates the system’s ability to simultaneously determine CVD risk in COVID-19 patients using the same CT scan. We developed and evaluated a deep learning-based approach to predict cardiac abnormalities from a CT scan of COVID-19 infected patients (**Figs 1B and 3**). A data set of 329 3D CT scans collected from 162 COVID-19 positive subjects and 167 uninfected healthy subjects has been used to evaluate the model. The data were divided into training and testing sets with 70% of collected data were used for training and 30% for testing. Constructed subset CT volume images were used as input in both the training and testing processes. The volume of CT scans was different for different images and hence they were resized to fit the input image size of each pre-trained 3D-CNN layers. Classification results were obtained from the 3D-CNN model with data augmentation [64]. Training data was randomly augmented in different angles and then the augmented data set was used to prepare the training model. With augmented training data, our model achieved 97.97% accuracy. However, our model has misclassified one cardiac image of a COVID-19 infected patient as normal heart and one cardiac image of a non-COVID-19 patient as CVD. When we checked the performance of our model by measuring various gold standard cardiac parameters for each CT scan, we observed that for some patients, despite having enough lung opacity, cardiac thoracic ratio (CTR) was less than 0.49 and pulmonary artery and aorta ratio (PA/A) was also less than 1. In our data set we observed one CT scan image of COVID-19 infected patient where CTR is less than 0.49 and at the same time PA/A is less than 1, that implies that in spite of having higher lung opacity the cardiac inflammation was very less for that covid-19 patients. On the other hand, the misclassification of one cardiac image of non-COVID-19 patient into a CVD may attributed to the fact that in this case the cardiac thoracic ratio (CTR) was greater than 0.49 and pulmonary artery and aorta ratio (PA/A) was also high. The measured CTR and PA/A parameters implies that CVD risk associated with this specific case, even though the subject confirmed to be COVID-19 negative. Thus, our model successfully diagnosed that CVD risk from CT scans of normal healthy subjects as well.

Studies based on 3D convolutions for 3D medical image analysis have been displayed instead of employing 3D spatial information as input in 2D classification algorithms. This method can capture 3D context along any axis.3D techniques are frequently preferred when a 3D context is required. The working mechanism of 3D CNN is very similar to 2D CNN, with the exception that the kernel movement is now three-dimensional, resulting in improved capture of relationships within the three dimensions and a difference in output dimensions after convolution (**Fig 3**). Harmon et al recently reported lung segmentation from 3D CT scans, using the 3D CNN model [65]. Valeriani et al reported a 3D CNN-based dystonia prediction [66]. By considering the diagnostic accuracy and performance on medical imaging we developed a proposed 3D-CNN model to predict cardiac abnormalities from chest CT images. At the same time, we rechecked the performance of 3D-CNN and validated against CT measured-cardiac parameters including (i) cardiac thoracic ratio (CTR), (ii) pulmonary artery to aorta ratio (PA/A), (iii) presence of calcified plaque and (iv) epicardial adipose tissue area to predict cardiac abnormalities in infected individuals.

#### Evaluation metrics

**Fig 5:**
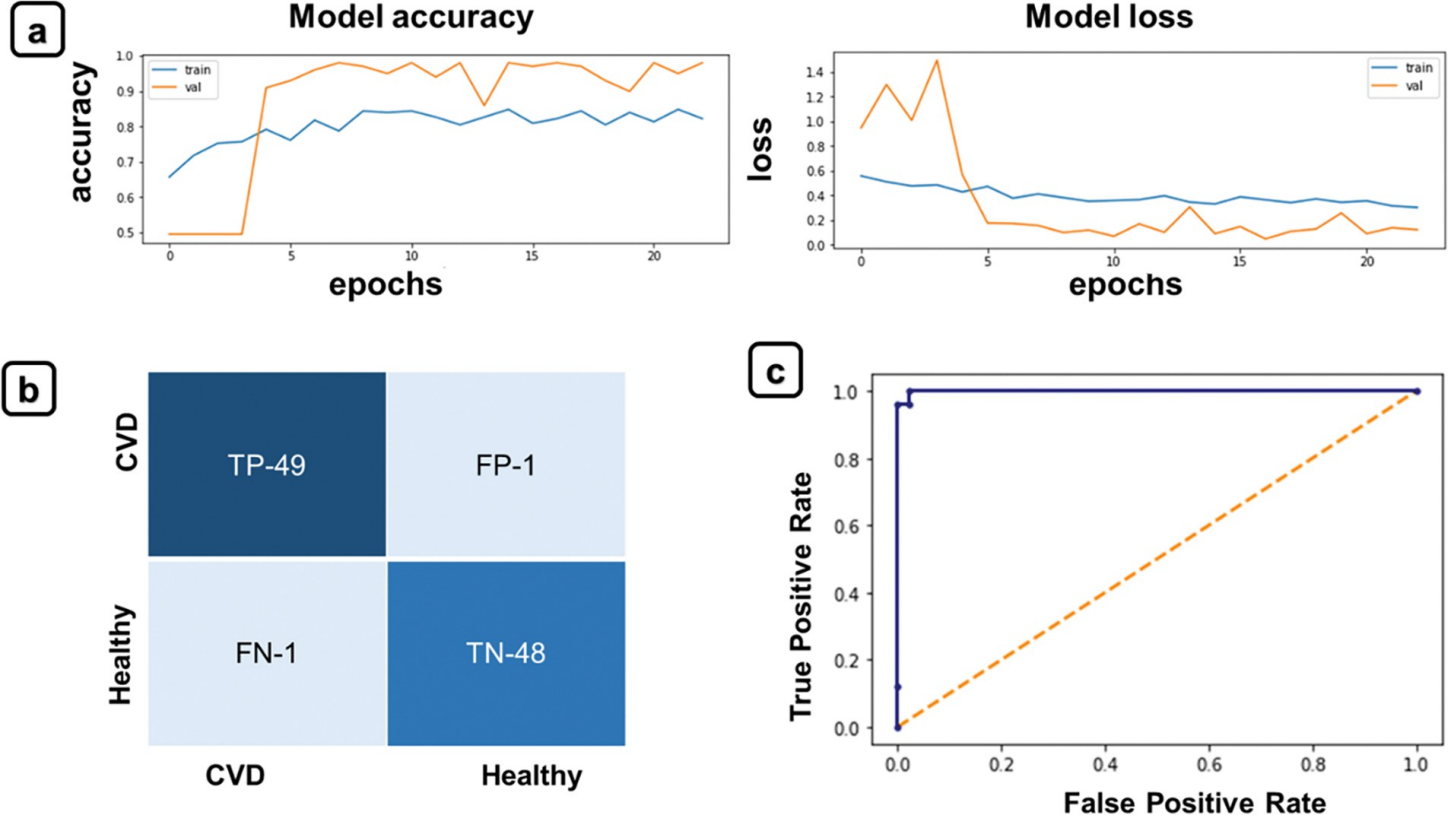
(a) Training and validation accuracy curve, and loss curve as achieved with augmented training data sets with 97% accuracy. (b) Confusion matrix and (c) ROC curve to evaluate the accuracy after 3D classification of the cardiac component from the CT scans of COVID-19 infected and uninfected healthy subjects.

We obtained accuracy and loss curves to evaluate the training performance of 3D-CNN model (**Fig 5A**). The loss and accuracy functions were found to be inversely proportional, as accuracy improved the loss decreased, and vice versa. On the other hand, accuracy, precision, recall, F1 score and ROC curve were reported to assess the classification result. Diagnostic accuracy for cardiac abnormality prediction is 97%, precision is 0.98, recall is 0.98 and the F1-score is 0.97. ROC curve and the confusion matrix are also represented in **Fig 5B and 5C** The sensitivity and the specificity of the model were found to be ~100% and 96.15% respectively. We thus demonstrated that our deep learning CNN model can predict COVID-19 associated CVD from CT scan with a high level of accuracy.


$$Precision=\frac{true\;positive}{true\;positive+false\;positive}$$

$$Recall=\frac{true\;positive}{true\;positive+false\;negative}$$

$$F1\;score=2\frac{Precision\times Recall}{Precision+Recall}$$

#### Probing the impact of COVID-19 on the cardiovascular system

**Fig 6:**
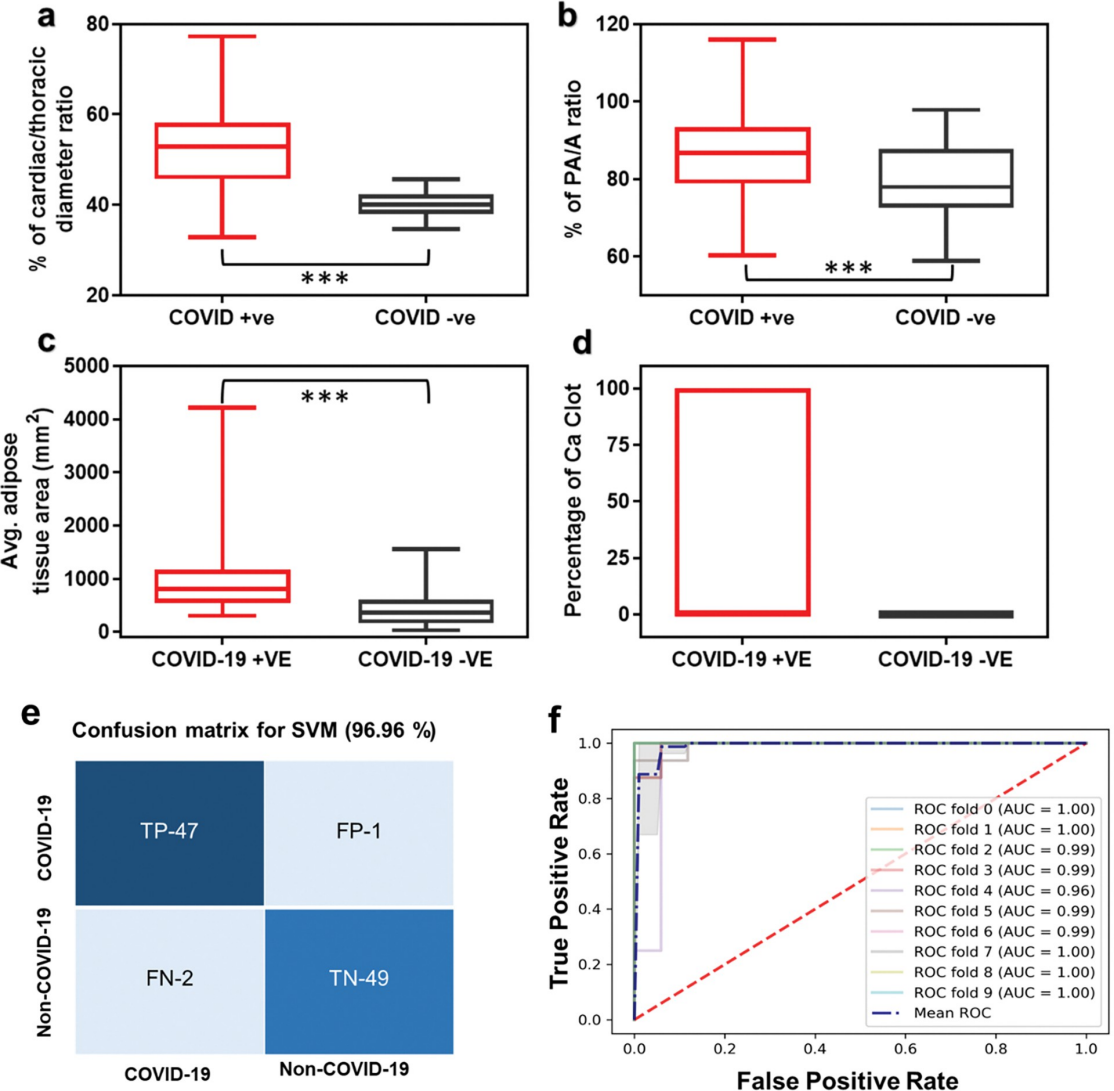
(a) Percentage of cardiac and thoracic diameter ratio, (b) pulmonary artery and aorta ratio; (c) average adipose tissue area and (d) percentage of calcium plaque are plotted for 162 COVID-19 positives and 167 COVID-19 negative patients. The percentage of each case is higher in positive cases than negative cases. The datasets were found to be highly statistically significant among each other: P < 0.0001 (***). (e) confusion matrix and (f) ROC-AUC curve for 10-fold cross validation performed on cardiac parameters.

Another important finding of the present study was the detailed understanding of cardiac health when infected with SARS-CoV-2 from CT measured cardiac parameters. Four cardiac parameters were extracted from the 3D CT images including, CTR, PA/A, observed calcified plaques, and mean epicardial adipose tissue area (**Fig 1C).** Of these parameters, CTR ≥ 0.49 is an indicator of cardiac enlargement since this factor is highly correlated with the higher opacity regions in the lungs [67]. Increased CTR is also considered as a sign of cardiac hypertrophy or cardiomegaly, which can cause shortness of breath, chest pain and may result in life-threatening abnormal heart rhythms or sudden death [56, 68]. The ratio of the diameter of the pulmonary artery (PA) to the diameter of the aorta (A) on CT images is associated with pulmonary artery enlargement or pulmonary hypertension [4, 57]. The PA/A ratio has been linked to changes in right heart structure and function, as well as pulmonary hemodynamics. These measurements can easily be derived from CT and associated with severe exacerbations of chronic obstructive pulmonary disease (COPD) [57, 69, 70]. Coronary artery calcified plaque (CAC) is, on the other hand, a powerful predictor of CVD events and mortality, both in general populations and high-risk patients with type 2 diabetes. Epicardial adipose tissue thickness is also associated with CVD risk [71, 72]. It is to be noted that the CT measured parameter CTR, PA/A and presence of calcified plaques were considered as gold standards for CVD risk predictors. All these gold-standard parameters for COVID-19 infected patients were calculated in this study and compared with the healthy subjects where we observed significant differences in values among individuals [4, 73]. The other CT measured parameter, *i*.*e*., mean epicardial adipose tissue thickness for COVID-19 patients, was also calculated which conveyed prognostic information about severe CVD risk factors [74]. We observed that increased CTR and PA/A are prevalent among severe COVID-19 patients. CT images of COVID-19 infected subjects with higher opacity regions and cases with significantly elevated CTR ≥ 0.49 were utilized for the deep learning-based CVD risk prediction compared to the healthy subjects. COVID-19 infected CT images were then labeled with higher CVD risk based on the abnormal CT measured cardiac parameters such as elevated CTR ≥ 0.49, PA/A ≥ 0.9 to 1, and an identified region for calcium plaques. An increased PA/A ≥ 1 was found in almost 56% of infected patients. However, the presence of calcified plaque was found in ~20% of COVID-19 infected patients, whereas the plaque was missing for uninfected healthy individuals in all cases. We also observed that the area of adipose tissue was significantly higher in COVID-19 infected patients in comparison to the healthy subjects. These measurements for COVID-19 individuals were compared with healthy subjects and represented graphically by box and whisker plots (**Fig 6A–6D)**. These plots were found to be quite statistically significant.

ML classifier SVM was then used to predict the impact of COVID-19 on the cardiac health of infected individuals compared to the healthy subjects from the aforementioned cardiac parameters. 70% of these CT measured parameters were used for training the ML classifier SVM and the model tested on a 30% test dataset. Once the training model was evaluated on an independent test dataset, it achieved 96.96% accuracy, with a precision value of 0.94 and recall of 0.97. This model obtained sensitivity and specificity of 96.08% and 97.92% respectively. The confusion matrix as obtained from the classification has been shown in **Fig 6E**. for further validation of the entire dataset and to estimate the robustness of the ML model, 10-fold cross-validation has been performed, where all the cardiac parameters for 329 subjects were split into 10 subsets, to evaluate the model performance one of which was used for validation while remaining 9 subsets were used as a training set. ML classification was performed using an SVM classifier. It turned out that after training ten times, only the SVM model achieved a 99% average ROC-AUC score and the curve for 10-fold cross-validation has been shown in **Fig 6F.** Thus, our results indicated a significant change of cardiac parameters for COVID-19 patients as compared to the healthy subjects. This is in corroboration of the data calculated above (**S13 and S14 Figs in S1 File**) which was also found to be highly statistically significant with *a p*-value less than 0.0001 in **Fig 6A–6D**.

## Conclusion

A series of technical breakthroughs have led to myriad new ways to use in diagnostic medical imaging. Following the trend, the onset of the COVID-19 pandemic also witnessed extensive evaluation of AI for lung CT images. The fundamental interest in CT is due to the high sensitivity of chest CT in diagnosing COVID-19 and its ability to predate a positive viral laboratory test. Based on chest CT imaging, the severity of the lung can be predicted, and the disease progression can be determined, which helps make treatment decisions. Several recent studies showed that CT examinations can unexpectedly detect lung abnormalities caused by COVID-19 which is performed in patients for other clinical indications presenting no respiratory complaints. In a pandemic situation, the hospitals are under much pressure with a high volume of admissions. Therefore, tools that can help us use CT for rapid triage of patients with plausible COVID-19 infection will be very useful. In summary, our study has proposed an ML pipeline for simultaneous detection of COVID-19 and its associated cardiovascular complications. The model, developed herein, was able to detect COVID-19 cases using a CT scan and differentiate them from other pneumonia cases and healthy subjects with 99.2% accuracy. Further, the deep neural network pipeline predicts the cardiac abnormalities associated with COVID-19 patients from the CT scan with 97.97% accuracy. Three CT-based cardiac parameters were used as gold-standard markers for the identification of COVID-19 associated CVD risk from CT images. We successfully created a deep learning model for automated classification and detection of COVID-19 pneumonia from non-COVID-19 cases and for assessing and predicting cardiac abnormalities in infected individuals. We focused on developing ML-based tools in our study. So, the emphasis was placed on utilizing secondary datasets from public image databases to develop the tools. Despite the benefits of integrating clinical data with CT images for improved and precise diagnosis, we could not implement this approach due to the constraints imposed by the dataset used. A follow-up study is warranted to explore this aspect and use primary datasets in which pertinent patient information will be integrated. We believe that the outcome of this project is significant, and the tools developed here will be clinically implemented in real-world practice.

## Supporting information

S1 File Contains the supporting figures (S1-S14 Figs) and supporting tables (S1-S3 Tables). (DOCX)

S2 File Calculated CT measured parameters [CTR, PA/A], basic demographic information [age, sex], and patients’ severity information of COVID-19 and healthy subjects. (XLSX)

S3 File Data Distribution of SARS-CoV, MERS-CoV and Pneumonia along with SARS-CoV-2 and healthy subjects. (XLSX)
