# Supplementary material for: Diagnosis of COVID-19 with simultaneous accurate prediction of cardiac abnormalities from chest computed tomographic images
Source: PLoS One. 2023 Dec 14;18(12):e0290494. doi: 10.1371/journal.pone.0290494 (PMC10721010; doi:10.1371/journal.pone.0290494)
Supplement: S1 File — (DOCX) [file pone.0290494.s001.docx]

**Supplementary information**

**Diagnosis of COVID-19 with Simultaneous Accurate Prediction of Cardiac Abnormalities from Chest Computed Tomographic Images**

Moumita Moitra,^1,2,¶^ Maha Alafeef,^1,2,3,4,¶^ Arjun Narasimhan,^1,&^ Vikram Kakaria,^1,&^ Parikshit Moitra^1,4^ and Dipanjan Pan*^,1,2,4,5,6^

^1^Center for Blood Oxygen Transport and Hemostasis, Department of Pediatrics, University of Maryland Baltimore School of Medicine, 670 W Baltimore St., Baltimore, Maryland 21201, USA

^2^Department of Chemical, Biochemical and Environmental Engineering, University of Maryland Baltimore County, 1000 Hilltop Circle, Baltimore, Maryland 21250, USA

^3^Biomedical Engineering Department, Jordan University of Science and Technology, Irbid 22110, Jordan

^4^Department of Nuclear Engineering, The Pennsylvania State University, University Park, Pennsylvania 16802, USA

^5^Department of Materials Science & Engineering, The Pennsylvania State University, University Park, Pennsylvania 16802, USA

^6^Huck Institutes of the Life Sciences, 101 Huck Life Sciences Building, University Park, PA 16802, USA

* Email of the corresponding author: [dipanjan@psu.edu](mailto:dipanjan@psu.edu)

¶ These authors contributed equally

& These authors also contributed equally

**Table of Contents Page number**

Figure S1 3

Figure S2 3

Figure S3 4

Figure S4 4

Figure S5 5

Figure S6 6

Figure S7 6

Figure S8 7

Figure S9 8

Figure S10 10

Figure S11 10

Figure S12 11

Figure S13 11

Figure S14 12

Table S1 13

Table S2 13

Table S3 14

Table S4 14

**Figures.**

**
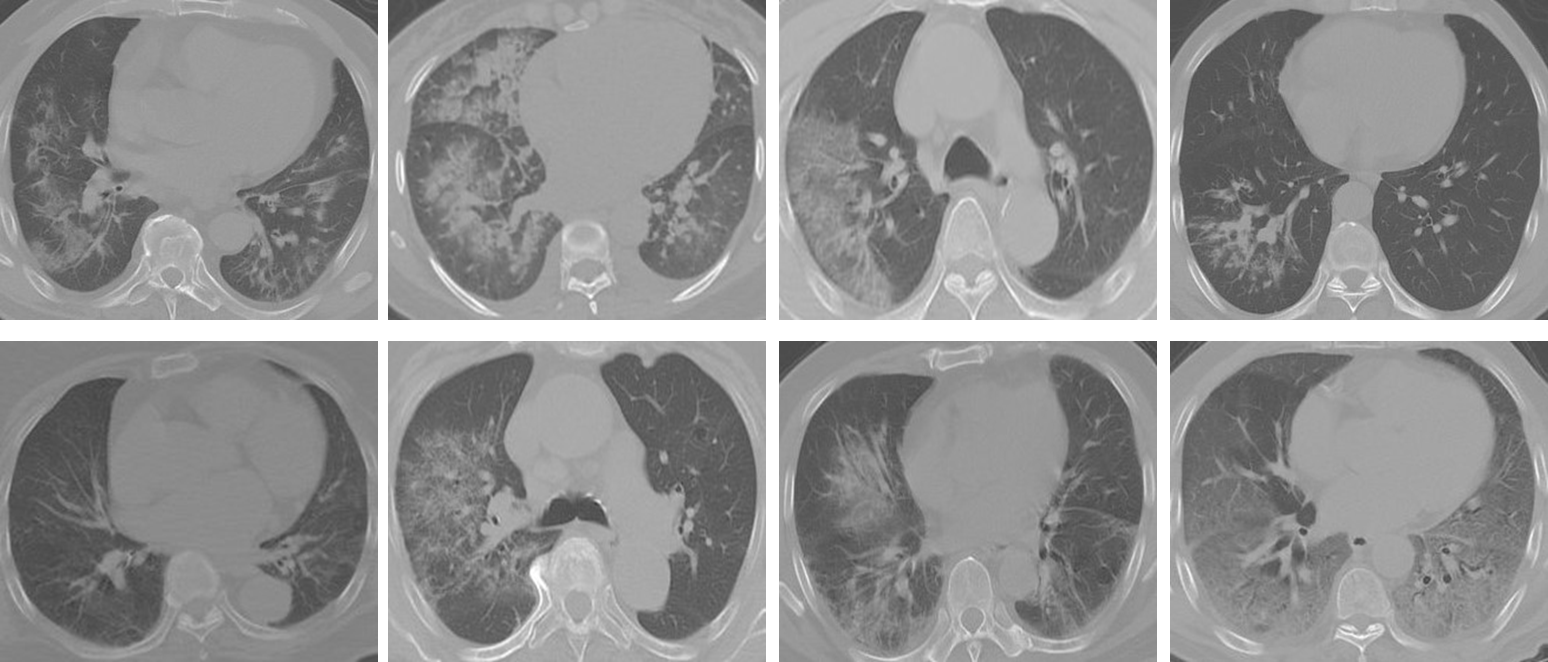
**

**Fig S1.** Representative CT images of COVID-19 associated CVD risk estimated subjects.

**
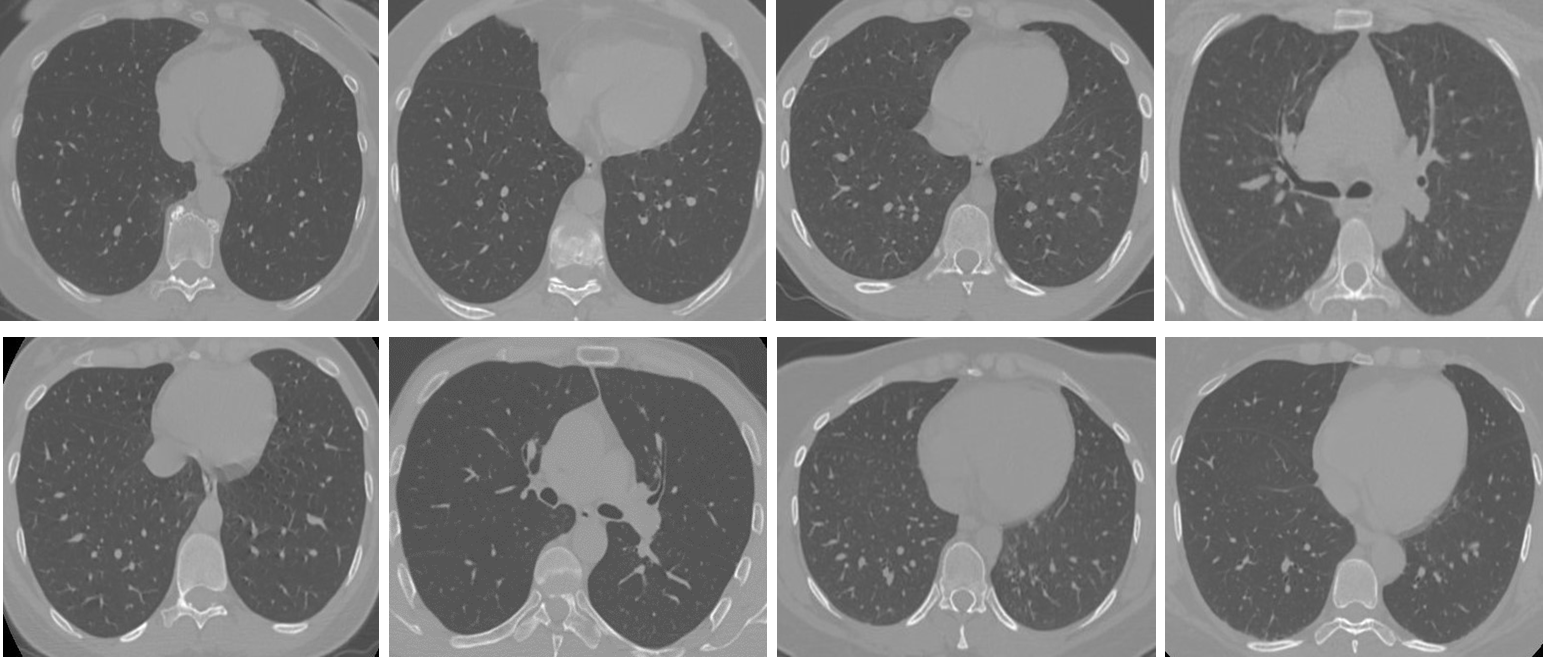
**

**Fig S2.** Representative CT images of healthy subjects.


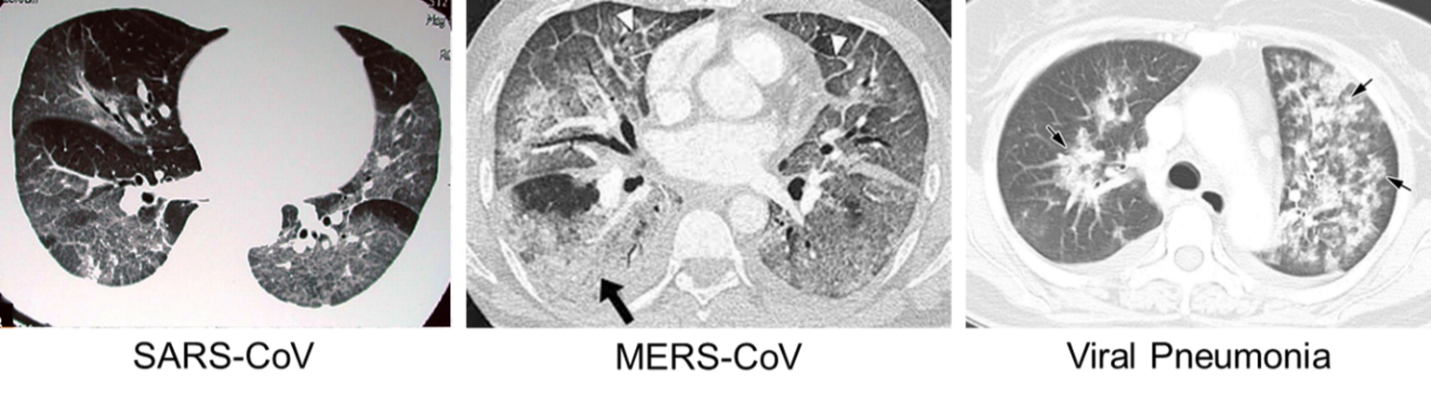
**Fig S3.** Representative images for SARS-CoV, MERS-CoV and viral pneumonia.


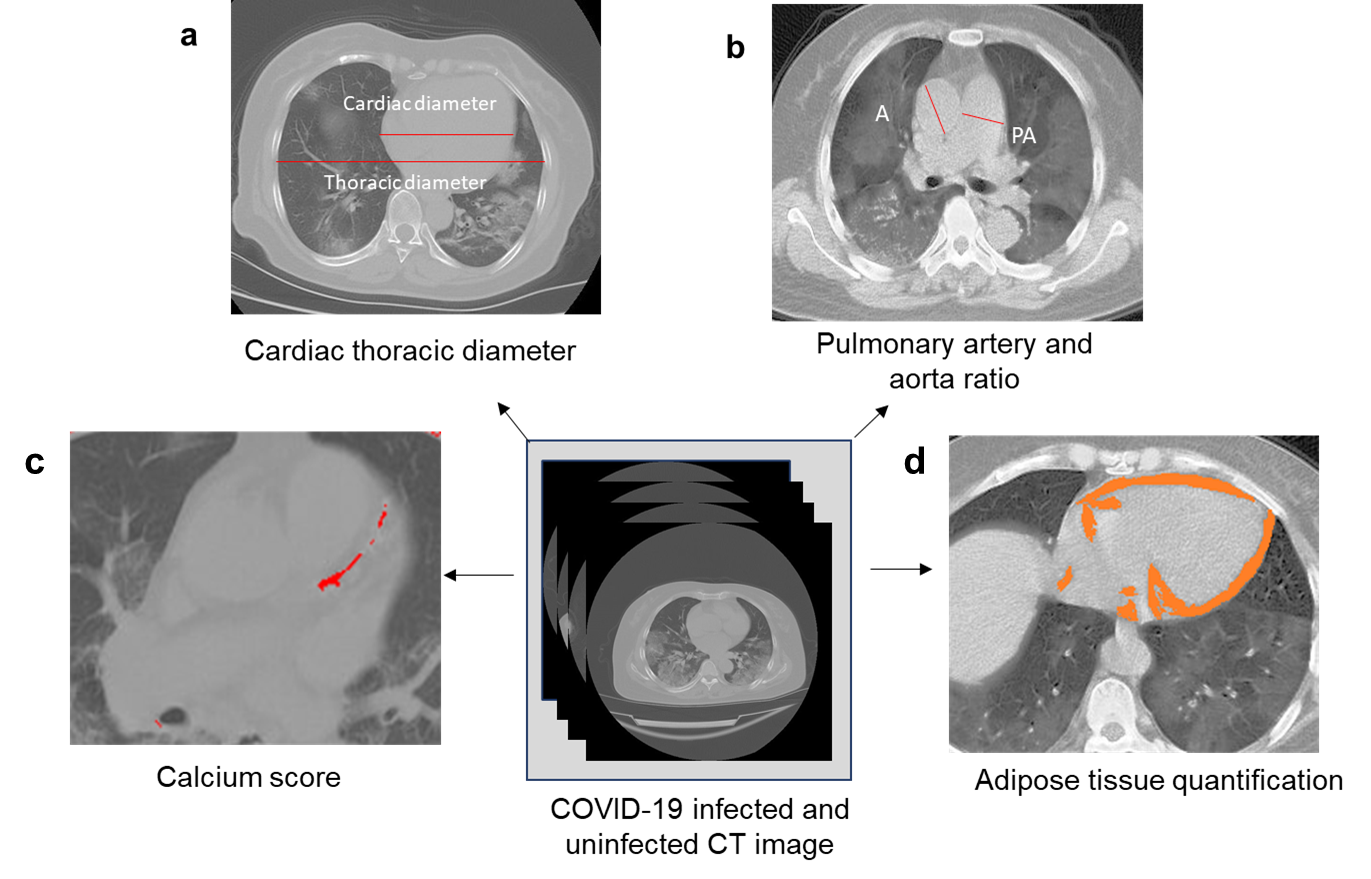


**Fig S4.** **CT measured cardiac parameters.** Four cardiac parameters were measured from CT scans of 162 COVID-19 infected and 167 uninfected healthy subjects. **(a)** Representative CT measured cardiac diameter and thoracic diameter ratio; **(b)** Representative CT measured pulmonary artery and aorta ratio PA/A; **(c)** Observed calcified plaque, calcium plaque was not present in a CT scan for healthy subjects; **(d)** Representative average epicardial adipose tissue area as calculated by thresholding.


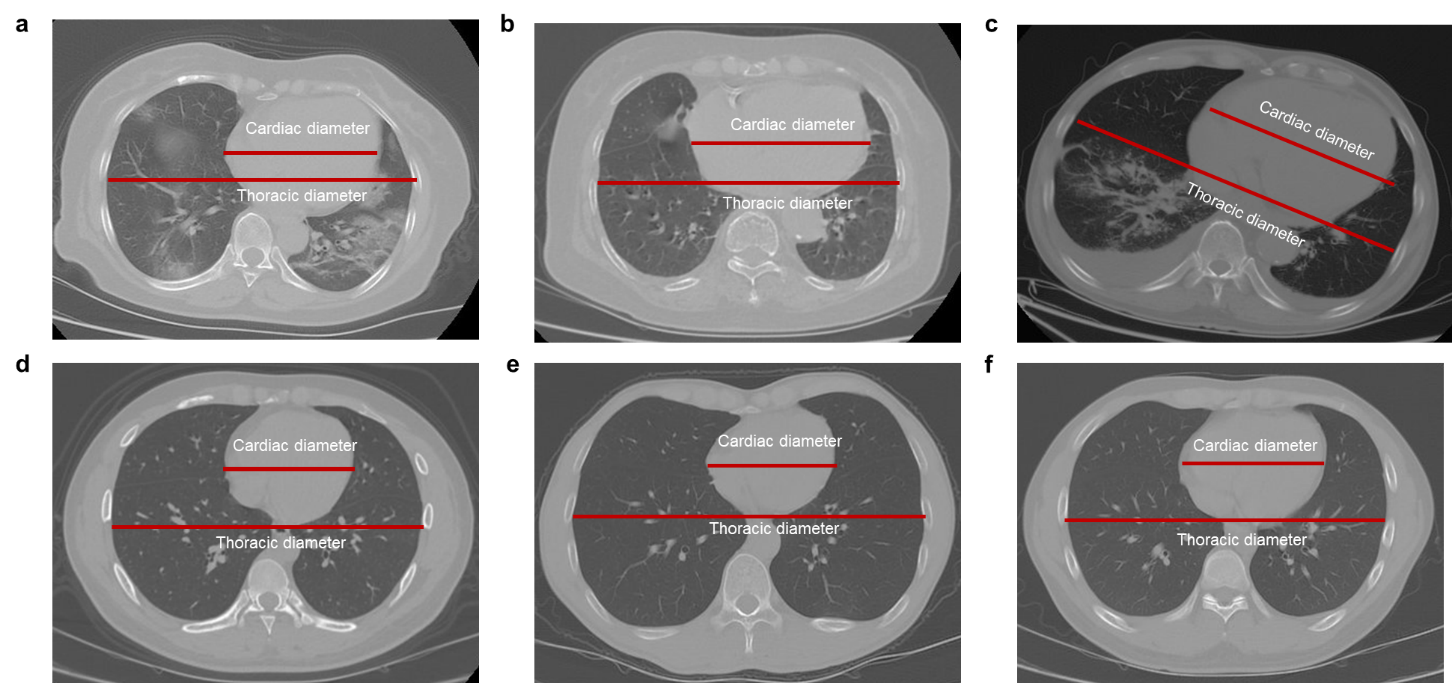


**Fig S5.** **Representative image of COVID-19 infected and uninfected patients:** CT images of (a-c) three COVID-19 infected patients and (d-f) three uninfected patients were used to measure the cardiac and thoracic ratio (CTR).


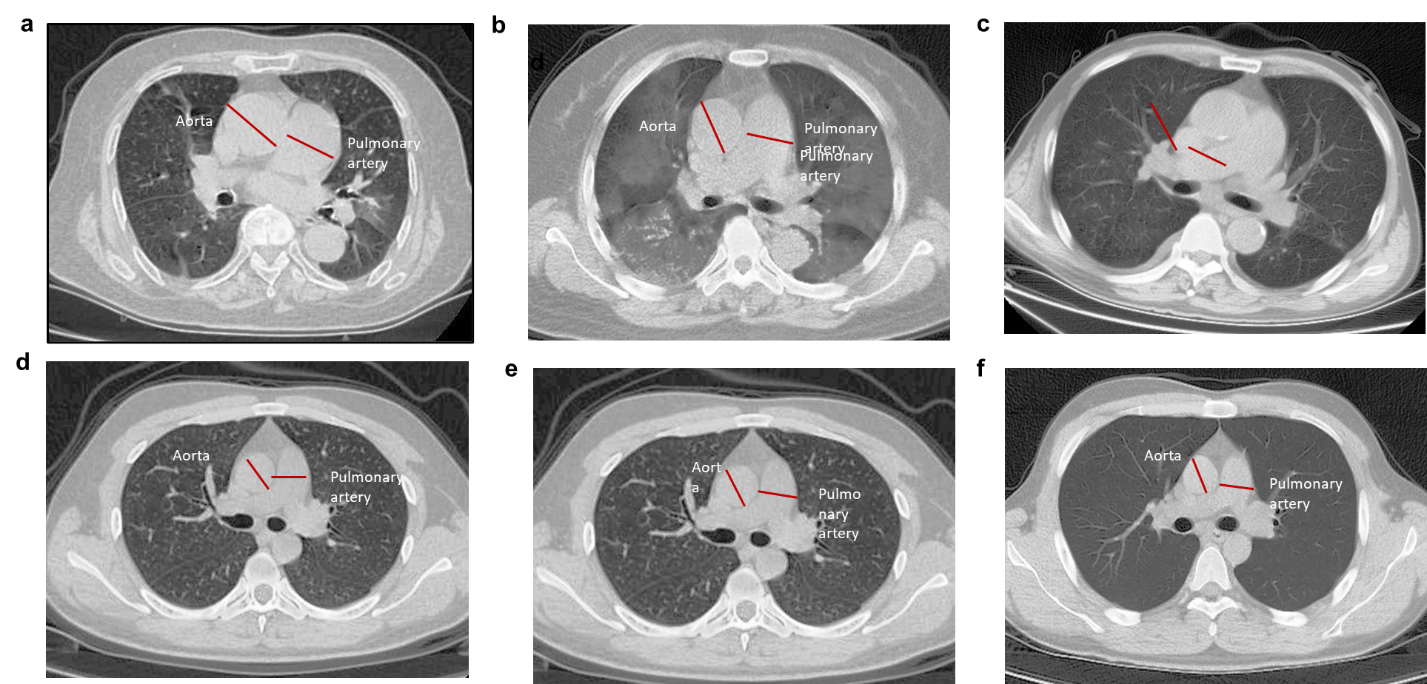


**Fig S6.** Pulmonary artery and aorta ratio (PA/A) was measured using representative CT images for (a-c) three COVID-19 infected patients and (d-f) three uninfected patients.


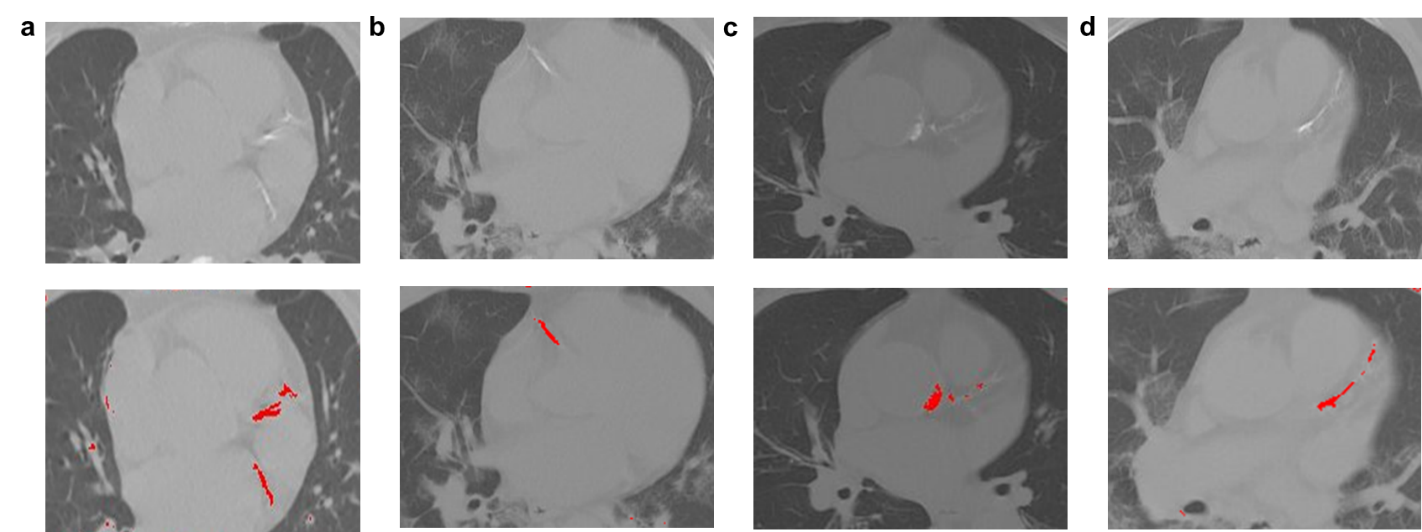


**Fig S7.** **Presence of calcified plaques in CT scans of COVID-19 infected patients:** four such cases have been shown in a-d. A calcium clot was not present in a normal patient's CT scan.


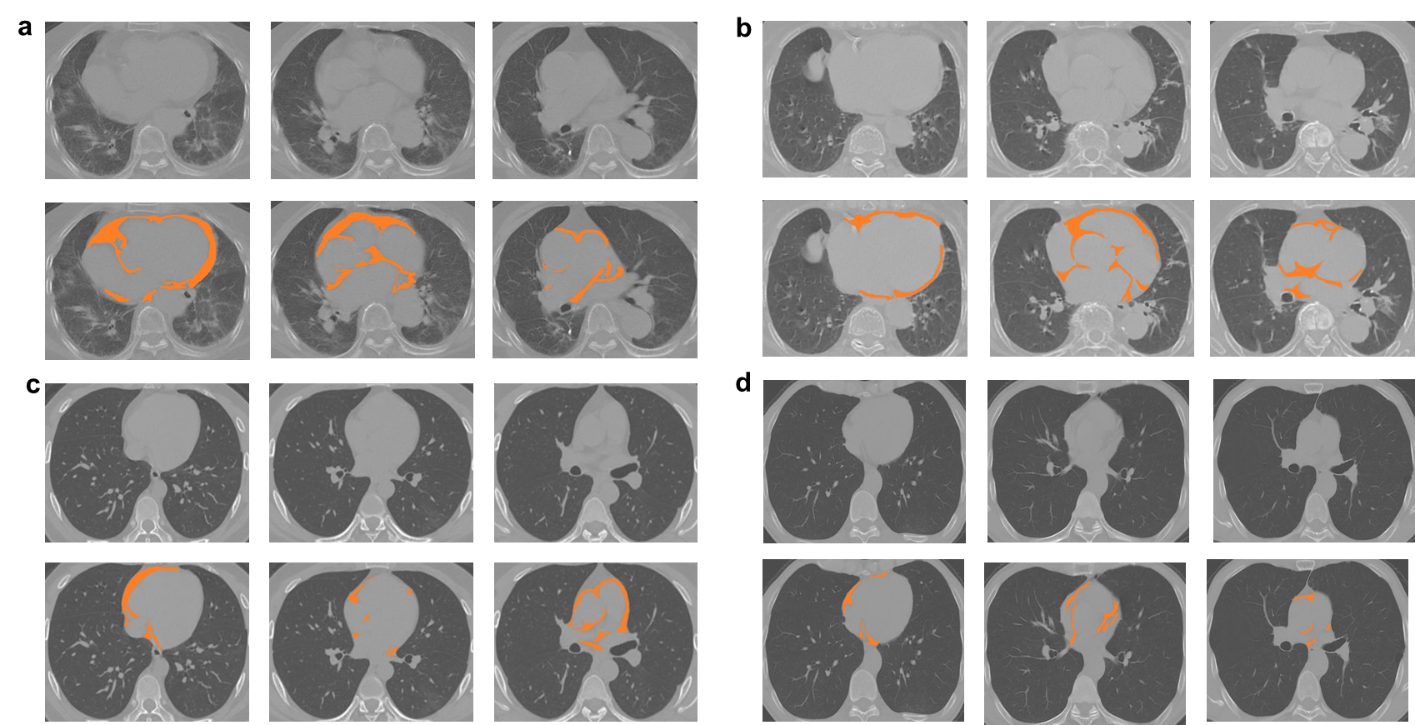


**Fig S8.** **Segmented Epicardial Adipose Tissue:** Three representative CT-image slices for (a, b) two COVID-19 infected and (c,d) two non-infected patients were used to measure epicardial adipose tissue area. We measured adipose tissue area from 3 slices of one 3D CT scan, one from the beginning where the heart is visible, the second from the center, and the last one from the slice where the heart section is ending. Adipose tissue area was then segmented by thresholding in ImageJ and calculated its average area.


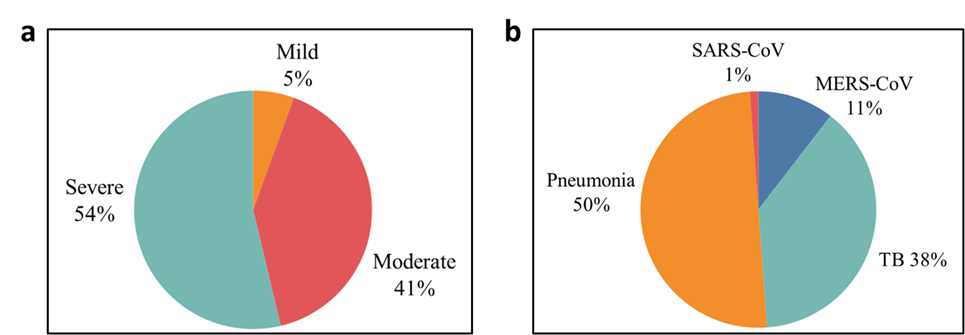


**Fig S9.** (a) Pie graph showing the severity of COVID-19 infected patients; (b) Pie graph showing distribution of pneumonia infected patient’s dataset other than SARS-CoV-2.


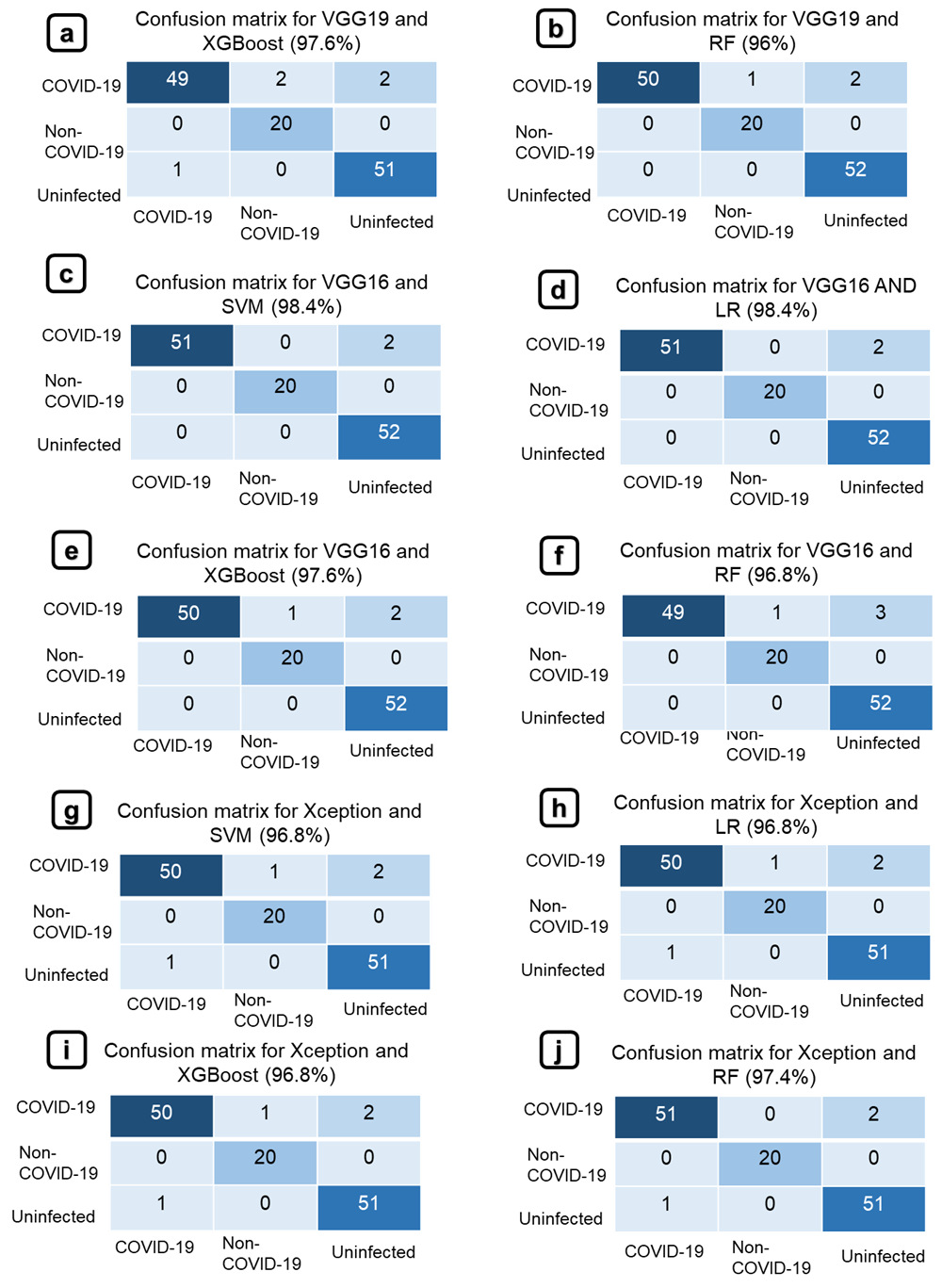


**Fig S10. Confusion matrix for all evaluated models used to distinguish COVD19 from other pneumonia and healthy subjects:** (a,b) Confusion matrix for the classification with pre-trained model VGG19 and machine learning classifiers XGBoost and random forest; (c,d,e,f) Confusion matrix for the classification with pre-trained model VGG16 and machine learning classifiers SVM, logistic regression, XGBoost and RF; (g,h,i,j) Confusion matrix for the classification with pre-trained model Xception and machine learning classifiers SVM, logistic regression, XGBoost and RF. Here we have classified the CT-scan images into three classes, COVID-19, other pneumonia, and uninfected individuals. Here a total of 415 images were used of which 290 were used for training and 125 were used for testing.


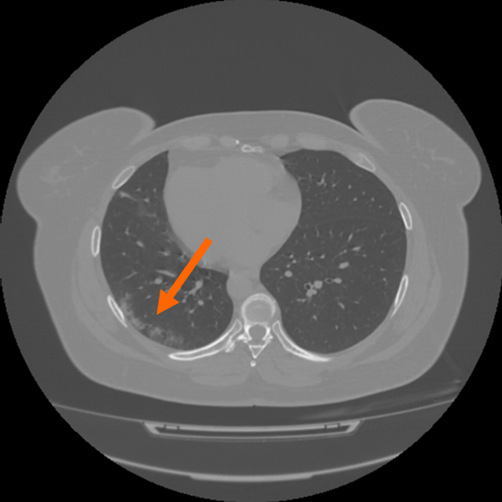


**Fig S11.** CT scan image of covid-19 infected patient misclassified as normal healthy subject.


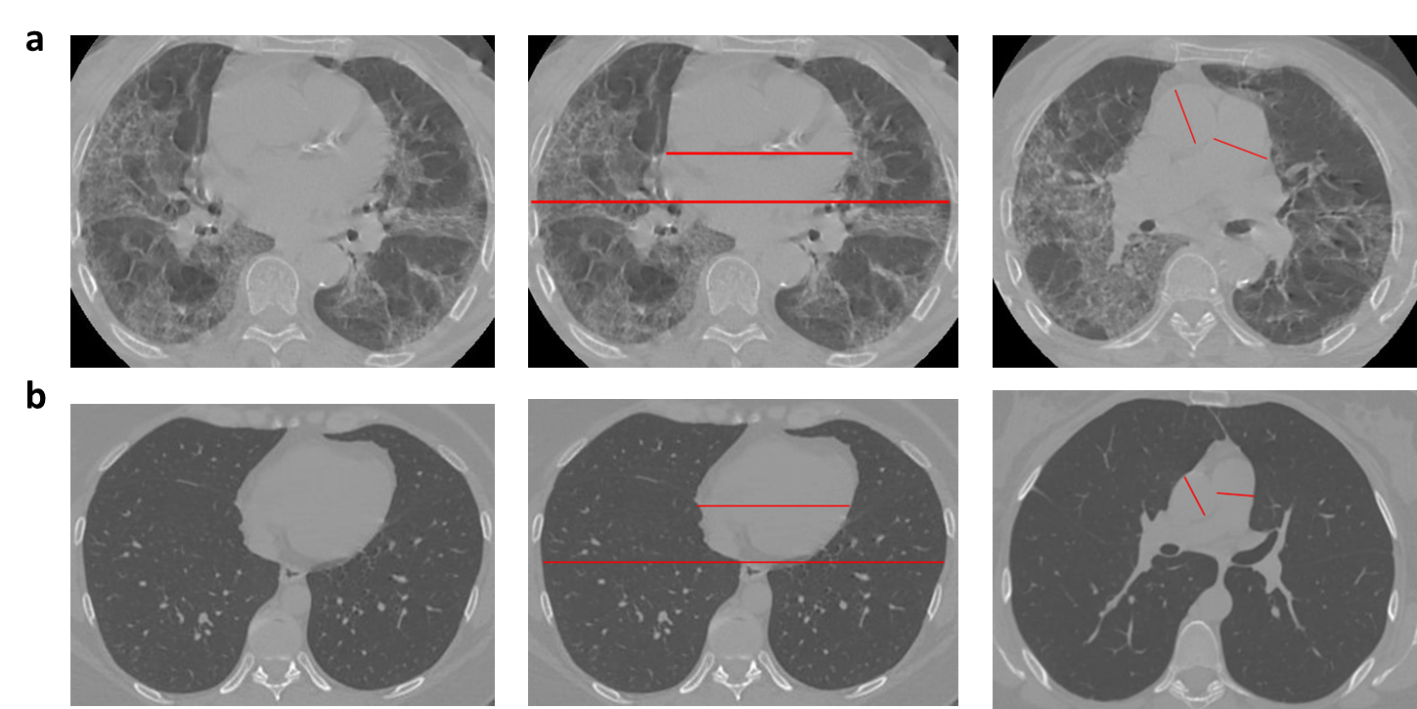


**Fig S12.** **CT measured parameter CTR and PA/A for COVID-19 infected patients and healthy subjects:** (a) CT scan images of COVID-19 infected patient with CVD, where we can see the CTR was greater than 0.49 and PA/A ratio was higher than 1. (b) CT scan images of non-COVID-19 patient with no CVD, where CTR and PA/A was comparatively less than the positive patients.

**Fig S13.** Percentage of cardiac and thoracic diameter ratio; pulmonary artery and aorta ratio for 162 COVID-19 infected and 167 uninfected subjects. The average percentage of cardiac/thoracic diameter for COVID-19 positive patients has been found to be higher than COVID-19 negative patients. The average percentage of PA/A ratio for COVID-19 positive patients was also observed to be higher than COVID-19 negative patients.

**Fig S14.** Average adipose tissue area for COVID-19 infected and uninfected healthy subjects has been plotted for 329 subjects’ data where 162 CT scans are from COVID-19 infected patients and 167 are from the uninfected subject. The average adipose tissue area has been always greater for positive COVID-19 cases than negative ones.

**Table S1.** Average of K-fold cross-validation score has been represented for all models.


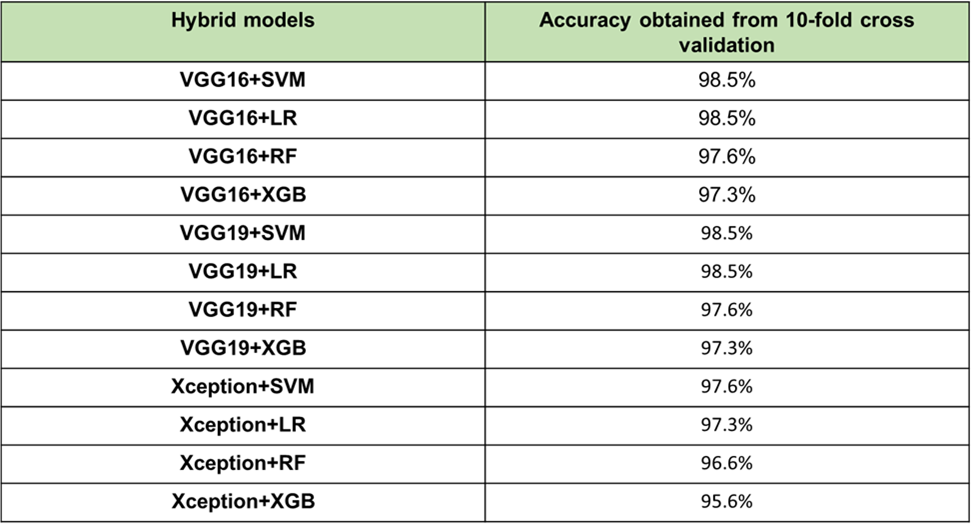


**Table S2.** Accuracy score, sensitivity and specificity has been represented for proposed models and the pretrained models as classifier used for COVID-19 classification from CT images.

| **Classification Models** | **Accuracy** | **Sensitivity** | **Specificity** |
| --- | --- | --- | --- |
| VGG16 |  |  |  |
| VGG19 |  |  |  |
| Xception |  |  |  |
| VGG16 + SVM |  |  |  |
| VGG16 + LR |  |  |  |
| VGG16 + RF |  |  |  |
| VGG16 + XGB |  |  |  |
| VGG19 + SVM |  |  |  |
| VGG19 + LR |  |  |  |
| VGG19 + RF |  |  |  |
| VGG19 + XGB |  |  |  |
| Xception + SVM |  |  |  |
| Xception + LR |  |  |  |
| Xception + RF |  |  |  |
| Xception + XGB |  |  |  |

100


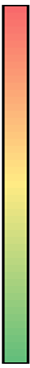


90

**Table S3.** Accuracy score, sensitivity and specificity has been represented for proposed models and the pretrained models as classifier used for COVID-19 classification from CT images.

**Table S4.** Calculated CT measured parameters [CTR, PA/A], basic demographic information [age, sex], and patients' severity information of COVID-19 and healthy subjects.

**Table S5.** Data Distribution of SARS-CoV, MERS-CoV and Pneumonia along with SARS-CoV-2 and healthy subjects.
